# PERSON-CENTERED CARE, BURNOUT, AND BARRIERS AMONG DIRECT CARE PROFESSIONALS: TARGETED TRAINING INTERVENTION 360

**DOI:** 10.1093/geroni/igz038.1868

**Published:** 2019-11-08

**Authors:** Lauren Stratton, Hannah Dannewitz, Jennifer Margrett, Mack Shelley, Linda Brown, Ann C Drobot

**Affiliations:** 1 Iowa State University, Ames, Iowa, United States; 2 Alzheimer's Association, Iowa Chapter, Cedar Rapids, Iowa, United States

## Abstract

Long-term care staff outcomes, such as job satisfaction and providing personalized care, are positively influenced by person-centered interventions. Implemented in eight facilities across Iowa, the Targeted Training Intervention 360 (TTI) program aimed to increase person-centered care among direct care professionals (DCP). Throughout the course of TTI, three waves of data were collected from DCPs regarding person-centered care (Person-Centered Care Assessment Tool; P-CAT) and feelings of burnout (Maslach Burnout Inventory; MBI). Analysis of variance tests were employed to identify significant differences in subscale scores across the three waves. Between waves one and two, results revealed significant increases in the P-CAT Extent of Personalizing Care (p=0.03) and Amount of Organizational Support subscales (p=0.001). Additionally, significant decreases from waves one and two were found in the MBI Emotional Exhaustion subscale (p=0.04). Between waves two and three, there were no significant changes in the P-CAT subscales; however, there was a significant increase in the MBI Emotional Exhaustion subscale (p=0.04). To supplement these findings, in wave three DCPs indicated barriers to implementing person-centered care, which included lack of time (49.0%), lack of experience (29.4%), and lack of administrative support (21.6%). Though there were no significant changes in P-CAT scores between the last two waves as well as barriers that must be addressed, DCPs described positive organizational and personal changes regarding person-centered care in the facility, including consistent staffing, using person-centered techniques in care, and individualized activities. Discussion focuses on ways to address barriers to person-centered care and sustain efforts in implementing change.

